# Systemic *Lonp1* Haploinsufficiency Mitigates Cardiac Mitochondrial Dysfunction Induced by Cardiomyocyte-Specific *Lonp1* Haploinsufficiency via Potential Inter-Organ Crosstalk

**DOI:** 10.3390/biom15081159

**Published:** 2025-08-13

**Authors:** Sakthijothi Muthu, Zinnia Tran, Ramasamy Saminathan, Pratikshya Shrestha, Sundararajan Venkatesh

**Affiliations:** Department of Physiology, Pharmacology and Toxicology, School of Medicine, West Virginia University, Morgantown, WV 26506, USA

**Keywords:** mitochondria, mitochondrial matrix, mitochondrial dysfunction, LONP1, protein quality control, heart, cardiac dysfunction

## Abstract

Efficient mitochondrial matrix protein quality control (mPQC), regulated by the mitochondrial matrix protease LONP1, is essential for preserving cardiac bioenergetics, particularly in post-mitotic cardiomyocytes, which are highly susceptible to mitochondrial dysfunction. While cardiac mPQC defects could impair heart function, it remains unclear whether such defects can be mitigated through inter-organ crosstalk by modulating mPQC in extra-cardiac tissues, a potentially valuable strategy given the challenges of directly targeting the heart. To investigate this, we examined two mouse models of *Lonp1* haploinsufficiency at young adulthood: a cardiomyocyte-specific heterozygous knockout (*Lonp1^CKO-HET^*) and a whole-body heterozygous knockout (*Lonp1^GKO-HET^*). Despite similar reductions in *Lonp1* mRNA expression in the hearts, *Lonp1^GKO-HET^* mice exhibited no cardiac dysfunction, whereas *Lonp1^CKO-HET^* mice showed mild cardiac dysfunction accompanied by activation of the mitochondrial stress response, including induction of genes such as *Clpx*, *Spg7*, *Hspa9*, and *Hspd1*, increased mitochondrial dynamics (*Pink1*, *Dnm1l*), reduced mitochondrial biogenesis, and compensatory upregulation of the mtDNA transcriptional regulator *Tfam*, all occurring without overt structural remodeling. These alterations were absent in *Lonp1^GKO-HET^* hearts. Our findings reveal a novel adaptive mechanism in which systemic mPQC deficiency can buffer mitochondrial dysfunction in the heart through inter-organ communication that is lost with cardiomyocyte-specific mPQC disruption. This study identifies systemic modulation of *Lonp1*-mediated mitochondrial stress pathways as a promising strategy to promote cardiac resilience through protective inter-organ signaling.

## 1. Introduction

Mitochondrial dysfunction contributes to heart failure, which remains one of the leading causes of heart disease [1,2,3]. Because mitochondria supply approximately 95% of ATP through oxidative phosphorylation (OXPHOS), even subtle mitochondrial dysfunction can disrupt the balance from adaptive to maladaptive remodeling [4]. Beyond ATP production, mitochondria also regulate calcium handling, redox signaling, apoptosis, and innate immunity, all of which demand functional mitochondria [5,6,7]. Such functional mitochondria are governed by various quality control processes, including proteostasis, fusion–fission dynamics, mitophagy, and apoptosis, which act as first responders to cardiac stress [7,8,9,10]. Hence, disturbances in mitochondrial function can lead to chronic compromised cardiac function over time, affecting ATP production.

Cardiomyocytes are uniquely vulnerable to intrinsic mitochondrial stress due to their terminally differentiated nature and limited regenerative capacity. At the same time, targeting these cells therapeutically is particularly challenging. In contrast, many extra-cardiac tissues possess robust regenerative and adaptive capabilities and may serve as potential reservoirs for initiating systemic protective responses [11,12]. Mounting evidence from metabolic and aging research suggests that mitochondrial perturbations in peripheral organs, such as skeletal muscle, liver, or adipose tissue, can trigger systemic stress responses, most notably mitochondrial stress that affects whole-body homeostasis and impacts distant organs [13,14,15]. This raises the intriguing therapeutic possibility of targeting other organs as an alternative to correcting mitochondrial defects within the diseased heart.

Mitochondrial matrix protein quality control (mPQC) is crucial for maintaining cardiac bioenergetics and cellular integrity, particularly in the post-mitotic myocardium. The heart, being a high-demand organ, relies heavily on well-orchestrated mitochondrial proteostasis mechanisms, which are primarily regulated by matrix proteases such as LONP1, along with other counterparts, including CLPXP, m-AAA, and iAAA proteases, to prevent the accumulation of damaged proteins and maintain oxidative phosphorylation [8]. Loss of mPQC function in cardiomyocytes could compromise mitochondrial efficiency, elevate oxidative stress, and lead to maladaptive remodeling and heart failure. However, emerging studies suggest that mitochondrial stress responses are not strictly cell-autonomous and may involve compensatory signaling across tissues [14]. While cardiac mPQC defects could impair heart function, it remains unclear whether such defects can be mitigated through inter-organ crosstalk, a process by which distant organs communicate via circulating metabolites, cytokines, or hormones to trigger adaptive or maladaptive responses. For example, mitochondrial stress in skeletal muscle or liver can release signaling molecules that influence cardiac mitochondrial function, offering a potentially valuable strategy given the challenges of directly targeting the heart. This raises the question: can the heart, when mPQC is impaired, benefit from mitochondrial stress responses initiated in distant tissues?

In this context, LONP1 which is a highly conserved ATP-dependent mitochondrial matrix protease, emerges as a critical regulator of systemic mitochondrial health. Beyond its role in degrading misfolded proteins, LONP1 modulates mitochondrial transcription, redox homeostasis, and stress signaling [8]. A growing body of evidence has implicated *Lonp1* haploinsufficiency as a critical contributor to mitochondrial and cardiac dysfunction. For instance, Venkatesh et al. (2019) demonstrated that reduced *Lonp1* expression sensitizes cardiomyocytes to ischemia–reperfusion injury, suggesting a key protective role for LONP1 in cardiac stress adaptation [16]. Similarly, De Gaetano et al. (2020) reported that *Lonp1* deficiency compromises mitochondrial ultrastructure and bioenergetics in embryonic fibroblasts, while Zhao et al. (2022) demonstrated that deletion of *Lonp1* in embryonic cardiac tissues leads to embryonic lethality due to arrested cardiac development [17,18]. Despite these significant advances, the consequences of *Lonp1* haploinsufficiency, specifically partial reductions in LONP1 activity, remain poorly understood, especially in young adult cardiomyocytes. Most studies to date have focused on either complete gene deletion or overexpression models, leaving a critical gap in understanding the physiological and pathophysiological relevance of more subtle, chronic deficits in *Lonp1* expression, which may better reflect disease or aging contexts. We hypothesized that moderate impairment of LONP1 in peripheral tissues might prime the organism to better tolerate mitochondrial dysfunction elsewhere, including in the heart. Such an adaptive preconditioning or “mitochondrial hormesis” model has been proposed in the context of aging and neurodegeneration, but its relevance to cardiac disease remains poorly defined [19,20].

To test this hypothesis, we employed two mouse models of *Lonp1* haploinsufficiency, a cardiomyocyte-specific heterozygous knockout (*Lonp1^CKO-HET^*) and a global heterozygous knockout (*Lonp1^GKO-HET^*), as homozygous global *Lonp1* deletion is embryonic lethal. Our study aimed to determine whether systemic modulation of mitochondrial proteostasis by partial deletion of *Lonp1* could indirectly protect the heart in settings of cardiac mPQC dysfunction. By comparing the mitochondrial stress signatures, cardiac function, and compensatory responses in these models, we reveal that systemic mPQC deficiency, but not cardiac-specific deficiency, shows adaptive mechanisms that safeguard the heart. These findings suggest a novel therapeutic paradigm in which targeting mitochondrial proteostasis in extra-cardiac tissues may offer cardioprotection in conditions of heart disease.

## 2. Materials and Methods

### 2.1. Reagents and Chemicals

All TaqMan assay probes were purchased from Bio-Rad, CA, USA, except for the 18S probe (Cat. # 4333760F, Applied Biosystems, Life Technologies, CA, USA) and the Tert probe (Cat. # 4458368, Applied Biosystems, Thermo Fisher, CA, USA) (Table S1). Chemicals were purchased from BioRad, Sigma, Applied Biosystem, Southern Biotech, Electron Microscopy Sciences, and GenScript unless otherwise mentioned.

### 2.2. Animal Models

All animal procedures were approved in accordance with the guidelines of the Institutional Animal Care and Use Committee (IACUC) of West Virginia University, USA (approval code 2203051893 on 6 April 2022). The C57BL/6 background whole-body heterozygous knockout *Lonp1^GKO-HET^* mice were obtained from Dr. Carlos López-Otín (Universidad de Oviedo, Spain), and described previously in Quiros et al., 2014 [21]. Cardiac-specific *Lonp1* heterozygous knock out (*Lonp1^CKO-HET^*) mice were generated using homozygous *Lonp1*-floxed mice (*Lonp1^flfl^*) (Obtained from Dr. Bin Lu, University of South China) crossed with mice expressing Cre recombinase under the endogenous α myosin heavy chain (α-MHC) promoter procured from Jackson Laboratory (Strain: 005657). Littermates of WT and Cre-WT mice (referred to Cre-Control) were used as control mice in all experiments, respectively. The young adult mice (~12-week-old) from both sexes were included in the study. Twelve-week-old mice were chosen to evaluate early mitochondrial and cardiac phenotypes before the onset of age-related changes, allowing for the detection of primary effects due to *Lonp1* haploinsufficiency without secondary influences. All the mice were housed (minimum 3 to maximum 5 per cage) and were fed with standard laboratory chow in a temperature- and light/dark cycle-controlled animal facility room with free access to food and water.

### 2.3. Genotype

Tail and Ear biopsies were collected from the litter to determine the genotype of the mice. DNA was extracted from the samples by lysing them at 75 °C for 10 min, followed by 5 min of incubation at 95 °C for enzyme activation using the kappa hot start mouse genotyping kit (Cat. # 07961804001, Roche Diagnostics, IN, USA). End-point polymerase chain reaction (PCR) was performed using the following primers synthesized by Eton Bioscience Inc., CA, USA. For *Lonp1^GKO-HET^*, we employed Forward (5′CCCTGACTGCAGAGATTGTGAA3′), (5′CAGGACATAGCGTTGGCTACC3′), and a common reverse primer (5′TTCAGTGCCAGTGCCTTAGAGT3′), whereas for the *Lonp1^CKO-HET^* we employed both flox; forward (5′GGATCACCCTGAGTTCCCAGTT3′) and reverse (5′CACCACCTATAGCAGGTGCGAA3′), and Cre; forward (5′GCCTGCATTACCGGTCGATGC3′), and reverse (5′CAGGGTGTTATAAGCAATCCC3′) primers. PCR products were amplified using a recommended protocol, and 2% agarose gel electrophoresis was used to separate the PCR products and determine the genotype. The WT genotype is indicated by the presence of a band at 200 bp, *Lonp1^GKO-HET^* bands at 200 bp plus 300 bp, αMHC Cre band at 501 bp, and *Lonp1^flfl^* bands at 473 bp plus 573 bp.

### 2.4. Transthoracic Echocardiographic Functional Assessment

Echocardiography on mice was performed by a single trained individual in a blindfolded approach in the WVU Animal Models and Imaging Facility. The mice were sedated with 1.5–2% isoflurane anesthesia via nose cone to ensure consistent and precise measurements of heart rate and function of left ventricle (LV). The echocardiogram images were acquired in parasternal long (PSLAX) and short axis (SAX) using a linear array transducer UHF57x (57 MHz) on the VevoF2 Micro-Ultrasound Imaging System (Visual Sonics, Toronto, ON, Canada). Both Brightness mode (B-mode) and Motion mode (M-mode) images of the heart were acquired at the level of papillary muscles to measure the ejection fraction (EF), fractional shortening (FS), stroke volume (SV), cardiac output (CO), left ventricular systolic, diastolic volume and diameter, anterior and posterior wall thickness. The echocardiographic analysis was performed utilizing a speckle tracking algorithm in VisualSonics Vevo Strain software 2.0 (Vevo F2, Visual Sonics, Toronto, ON, Canada) by an individual blindfolded to the mouse identifier. The endocardium and epicardium were tracked and marked up via speckle tracking and these segments were analyzed for at least three to five consecutive cardiac cycles to define the highest value achieved per parameter and averaged to give a comprehensive perspective of the heart. Raw values are provided in Table S2.

### 2.5. Histological Analysis of Cardiac Morphology

Mice were weighed and euthanized by cervical dislocation. The hearts were excised, weighed, rinsed in 1× ice-cold PBS, and dry blotted. The excised heart tissue was sliced horizontally using a Zivic Mouse Heart Slicer Matrix (Cat. # HSMS001-1, Zivic Instruments, PA, USA), enabling precise tissue region, and then fixed in a 10% Formalin solution (Sigma Aldrich). Subsequently, the tissues were hydrated and dehydrated in graded alcohol solutions, cleared with xylene, and embedded in paraffin wax. The paraffin blocks of the hearts were cut at 5 µm, deparaffinized in descending graded alcohol, and stained with hematoxylin and eosin (HE), and Masson’s Trichrome. Collagen volume fraction (CVF) was quantified by analyzing Masson’s Trichrome-stained heart sections using FIJI (ImageJ 1.54f, Fiji distribution version 2.15.0, National Institute of Health, USA) software. For each sample, multiple fields were randomly selected across the left ventricular myocardium. The collagen-stained area (blue) and total tissue area were quantified by applying a standardized color thresholding algorithm that selectively isolates blue pixels corresponding to collagen matrix, as distinct from the red-stained muscle fibers. CVF was then calculated as the ratio of collagen-stained area to total tissue area, and the mean CVF value from multiple fields was averaged per sample. These per-sample averages were used for statistical comparison across groups and expressed as a percentage of total tissue area. This method enables objective and reproducible quantification of myocardial fibrosis. Images were acquired at 10× and 40× magnification using the MIF Olympus slide scanner (Both Brightfield and fluorescence modes). Raw values are provided in Table S2.

### 2.6. Gene Expression Assay by RT-PCR

The experimental mice heart tissues were snap frozen in liquid nitrogen, ground to a fine powder employing pre-chilled mortar, followed by homogenization, and total RNA was extracted using the Qiagen RNeasy mini kit for fibrous tissue (Cat# 74704, Qiagen Inc.). The concentration of RNA (ng/µL) in each sample was measured using a Denovix 11 series Spectrophotometer. The RNA (100–500 ng) was utilized to synthesize cDNA by employing an iScriptTM cDNA synthesis kit from BioRad (Cat #170 8891), followed by cDNA quantification. qRT-PCR was performed employing TaqMan-based gene expression assays for each gene target as listed in Table S1. The relative fold expression was normalized against 18S and quantified using the ΔΔCt method. The list of TaqMan gene expression assays used in this study is provided in Table S1, and the corresponding raw Ct and ΔΔCt values used for calculating relative expression are provided in Table S2.

### 2.7. Determination of Mitochondrial DNA Content

Relative levels of mitochondrial DNA copy number (mtDNA-CN) from the heart tissues were measured using a real-time qPCR assay. Genomic DNA was isolated from mouse heart tissue following the modified simple DNA isolation method described in Muthu et al., 2025 [22]. 100 ng of the isolated genomic DNA was employed to quantify relative mtDNA content by amplifying both the *mt-Co1* gene and the nuclear *Tert* gene (Table S1). Amplification was performed using BioRad Universal PCR Master Mix (Cat#1725134, BioRad, CA, USA). The qPCR conditions included an initial denaturation at 95 °C for 10 min, followed by 40 cycles of denaturation at 95 °C for 15 s and annealing/extension at 60 °C for 1 min. All reactions were carried out in triplicate. The relative fold quantitation of the mtDNA copy number was calculated using the ΔΔCt method. Raw values are provided in Table S2.

### 2.8. Mitochondria Isolation from the Whole Heart

Hearts were harvested, squeezed to remove blood, and rinsed in ice-cold PBS. Then, mitochondrial isolation was performed at 4 °C using buffer 1 [100 KCL mmol/L, 50 MOPS mmol/L, 5 MgSO_4_·7H_2_O mmol/L, 1 EGTA mmol/L, and 1 ATP mmol/L (PH 7.4)] at a 1:10 (weight: volume) ratio to homogenize the heart tissue. The samples were centrifuged at 700× *g* for 10 min, followed by the collection of the supernatant and subsequent centrifugation at 10,000× *g*. The pellet was washed in buffer 1 and centrifuged twice at 10,000× *g*. The precipitated pellet from 700 g was further processed in buffer 2 [100 KCL mmol/L, 50 MOPS mmol/L, and 0.5 EGTA mmol/L (PH 7.4)], and digested in trypsin (5 mg/g) for 10 min, then the digestion was stopped by adding protease inhibitor cocktail and centrifuged at 700× *g* for 10 min, the supernatant was collected and recentrifuged at 10,000× *g* for 10 min. Mitochondria from the initial supernatant and digested pellets were combined in a sucrose buffer that contains 220 sucrose mmol/L, 70 mannitol mmol/L, 10 Tris-HCL mmol/L, and 1 EDTA mmol/L (PH 7.4). The further purification of total mitochondria was performed using a sucrose gradient of 23%, 15%, 10%, and 3% Percoll solution and centrifuging in a Beckman Optima MAX-XP Ultracentrifuge (Beckman Coulter, Fullerton, CA, USA) at 32,000× *g* for 8 min and then the final mitochondrial isolation was stored in KME buffer [KCL 100 mM, MOPS 50 mM, and EGTA 0.5 mM (PH 7.4)]. The final pellet of mitochondria was used for mitochondrial ETC complex activity analysis.

### 2.9. Enzyme Activity of Electron Transport Complexes I–V

The total protein concentration was determined by the BCA method from isolated mitochondria. The ETC complexes I, II, III, and IV, activities were measured using protein homogenates as described in Kunovac et al. 2021 [23]. Briefly, the activities of ETC complexes I to IV were determined by measuring the oxidation of NADH at 340 nm in the presence of decyl ubiquinone and rotenone (I), 2,6-dichlorophenolindophenol (DCPIP) reduction at 600 nm (II), cytochrome C reduction at 550 nm (III), and oxidation of reduced cytochrome c at 550 nm (IV), respectively. The complex V activity was determined by measuring oligomycin-sensitive ATPase activity through the pyruvate kinase and phosphoenolpyruvate pathway. The final values were represented as unit/nanogram or milligram of protein (I–V), where unit = nanomoles of oxidized substrate (minute^−1^). Raw values are provided in Table S2.

### 2.10. Statistical Analysis

The significant differences between control and *Lonp1* haploinsufficiency groups of both *Lonp1^GKO-HET^* and *Lonp1^CKO-HET^* models were individually compared using the unpaired Student’s *t*-test using GraphPad Prism software version 10.4.2, and *p* < 0.05 was considered statistically significant. Data are expressed as mean ± standard error of the mean.

## 3. Results

### 3.1. Cardiac-Specific but Not Global Lonp1 Haploinsufficiency Increases Heart Weight

**Figure 1:**
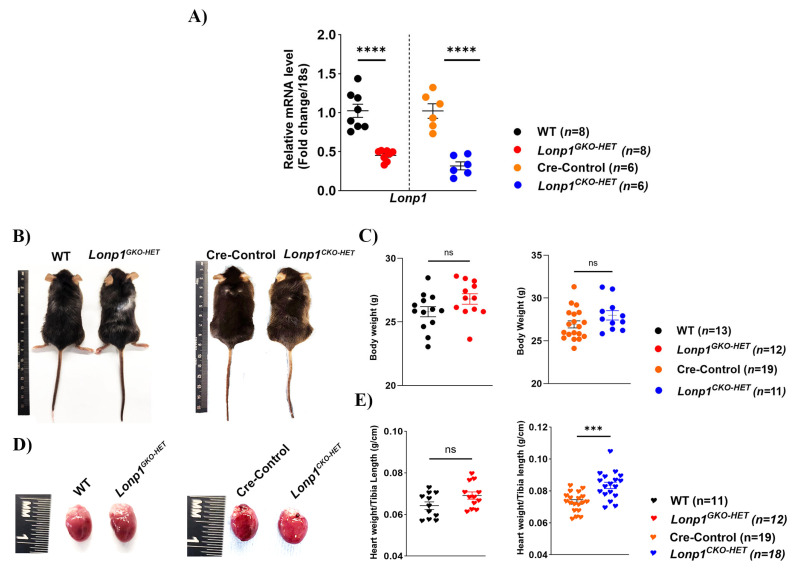
Effect of both systemic and cardiac-specific *Lonp1* haploinsufficiency on body and heart morphology. (**A**) A significant reduction in *Lonp1* expression is observed in both heterozygous models compared to their respective controls, confirming the efficiency of the partial knock-out in the groups as determined by RT-PCR analysis of *Lonp1* mRNA levels. (**B**) Representative dorsal views of male global heterozygous mice (*Lonp1^GKO-HET^*) and their wild-type (WT) littermates, and cardiomyocyte-restricted heterozygous mice (*Lonp1^CKO-HET^*) and their Cre-Control littermates and (**C**) corresponding scattered dot plots of their body weight. (**D**) Representative excised whole heart images of *Lonp1^GKO-HET^* and their wild-type (WT) littermates, and *Lonp1^CKO-HET^* and their Cre-Control littermates and (**E**) corresponding scattered dot plots of their heart-weight-to-tibia-length ratio (HW/TL). Values are represented as mean ± S.E.M. ns represents non-significant, **** *p* < 0.0001, *** *p* < 0.001 is considered significant by Student’s *t*-test.

At 12 weeks of age, global *Lonp1^GKO-HET^* and cardiomyocyte-specific *Lonp1^CKO-HET^* mice were phenotypically indistinguishable from their respective control groups regarding overall body weight. A significant ~50% reduction in *Lonp1* mRNA expression is observed in both heterozygous models compared to their respective controls, confirming the efficiency of the partial knock-out in these groups (Figure 1A). As expected, male mice weighed more than their female littermates (Figure S1); however, no significant differences in body weight were observed compared to controls in either group (Figure 1B,C). However, the *Lonp1^CKO-HET^* mice displayed a significantly increased heart weight comparable to Cre-Control hearts, while the *Lonp1^GKO-HET^* mice exhibited a modest but not significant increase in heart weight relative to their wild-type control hearts (Figure 1D,E). These findings suggest that the cardiac-specific partial loss of *Lonp1* within cardiomyocytes may be more vulnerable to the loss of *Lonp1*, while systemic loss of *Lonp1* may be compensatory.

### 3.2. Cardiomyocyte-Specific Lonp1 but Not Global Lonp1 Haploinsufficiency Show Mild Cardiac Dysfunction

**Figure 2:**
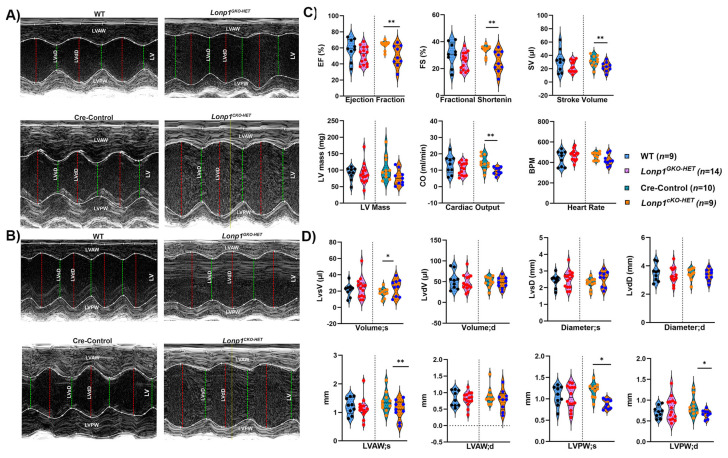
Echocardiographic assessment of cardiac function in both *Lonp1* haploinsufficiency groups. Representative M-mode (parasternal short-axis) from male (**A**) and female (**B**) *Lonp1^GKO-HET^*, *Lonp1^CKO-HET^*, and their respective control littermates. Green and red cursors delineate systolic (s) and diastolic (d) phases of the left ventricle. (**C**,**D**) Quantitative analysis of key functional parameters, including ejection fraction (EF), fractional shortening (FS), stroke volume (SV), cardiac output (CO), left-ventricular mass (LV mass), systolic volume and diameter, and anterior and posterior wall thickness obtained from VevoF2 Micro-Ultrasound Imaging System. LVsD; Left Ventricular systolic Diameter, LVdD; Left Ventricular diastolic Diameter, LVAW; Left Ventricular Anterior Wall, LVPW; Left Ventricular Posterior Wall, LV; Left Ventricular chamber. Values are represented as mean ± S.E.M. * *p* < 0.05, ** *p* < 0.01 is considered significant by Student’s *t*-test.

High-resolution transthoracic echocardiography performed at 12 weeks (Figure 2A,B) showed that *Lonp1^CKO-HET^* mice showed a significant reduction in left-ventricular ejection fraction (EF), fractional shortening (FS), stroke volume (SV), cardiac output (CO), and left-ventricular mass (LV mass) when compared to their Cre-Control littermates (Figure 2C). In addition, the left ventricular systolic volume and diameter showed a significant increase, accompanied by a significant reduction in left ventricular systolic and diastolic anterior and posterior wall thickness (Figure 2D). Conversely, global heterozygous (*Lonp1^GKO-HET^*) hearts exhibited modest downward trends in ejection fraction (EF) and fractional shortening (FS), with little change in systolic and diastolic posterior wall thickness compared to wild-type animals (Figure 2C,D). The heart rate remained comparable in both groups (Figure 2C). Collectively, these data indicate that partial reduction of *Lonp1* in a cardiomyocyte-specific manner impairs baseline systolic function in young adult mice, whereas systemic heterozygosity does not measurably compromise baseline systolic function in young adult mice.

### 3.3. Assessment of Cardiac Remodeling in Lonp1-Haploinsufficient Mice

**Figure 3:**
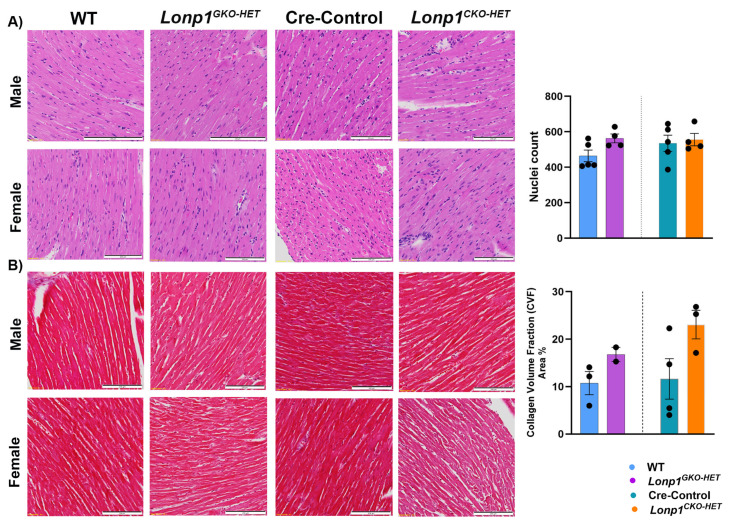
Histological evaluation of cardiac remodeling in both global and cardiomyocyte-specific *Lonp1*-haploinsufficient mice. (**A**) Representative HE-stained heart sections from male and female global heterozygous (*Lonp1^GKO-HET^*), wild-type (WT), Cre-Control, and cardiomyocyte-specific heterozygous (*Lonp1^CKO-HET^*) mice. The right panel shows the number of nuclei per high-power field. (**B**) Representative Masson’s trichrome-stained heart sections from male and female *Lonp1^GKO-HET^*, WT, Cre-Control, and *Lonp1^CKO-HET^* mice. The right panel shows Collagen Volume fraction (MT): collagen-positive area (% of total tissue). Images are shown at 40× (scale bar = 200 µm). Data represent a mean ± S.E.M. Statistical analysis by unpaired Student’s *t*-test showed no significant differences between control and corresponding *Lonp1* haploinsufficient mice.

To evaluate whether partial loss of *Lonp1* affects myocardial structure, we performed histological analyses of transverse heart sections from 12-week-old male and female mice across four genotypes: WT, *Lonp1^GKO-HET^*, Cre-Control, and *Lonp1^CKO-HET^*. Haematoxylin and eosin (HE) staining revealed preserved global myocardial architecture in WT, and *Lonp1^GKO-HET^* hearts across sexes, with compact and uniformly aligned cardiomyocytes and no signs of inflammatory infiltration or cellular degeneration. In contrast, *Lonp1^CKO-HET^* hearts, particularly those from male mice, exhibited a trend toward mild myocardial remodeling, characterized by subtle myocyte irregularity, increased variability in nuclear alignment, and a slightly more heterogeneous tissue appearance. In *Lonp1^CKO-HET^* hearts, a trend toward localized, mild perivascular and interstitial collagen accumulation was observed in males. Collectively, these results suggest that cardiac-specific haploinsufficiency of *Lonp1* may initiate early signs of structural remodeling. However, these changes are more of trends rather than definitive pathological alterations. These trends were less prominent in female *Lonp1^CKO-HET^* hearts, suggesting a potential sex-dependent difference in the structural response to cardiac-specific *Lonp1* haploinsufficiency (Figure 3A and Figure S2). To assess fibrotic remodeling, we performed Masson’s trichrome staining on matched heart sections. *Lonp1^GKO-HET^* hearts exhibited minimal collagen deposition, consistent with normal myocardial homeostasis, compared to their wild-type (WT) littermates. In *Lonp1^CKO-HET^* hearts, trichrome staining revealed localized, mild perivascular and interstitial collagen accumulation in males, while female hearts showed no significant fibrosis compared to their Cre-Control littermates (Figure 3B and Figure S2). Despite these observations, overall collagen deposition remained low, and quantitative collagen fraction analysis did not reveal statistically significant differences across groups. Collectively, these results indicate that cardiac-specific haploinsufficiency of *Lonp1* is sufficient to initiate early structural remodeling without triggering widespread fibrosis at 12 weeks of age. The absence of overt fibrotic changes suggests that these alterations may represent an adaptive response to early mitochondrial stress rather than irreversible pathological remodeling, with potential modulation by sex-specific factors.

### 3.4. Cardiac-Specific Lonp1 Haploinsufficiency Induced Mitochondrial Stress Response in the Heart, in Contrast to Global Lonp1 Haploinsufficient Mice

As transcript levels are often more reliable indicators than proteins under stress induction conditions because transcriptional responses occur rapidly and directly reflect gene activation before translational or post-translational modifications influence protein abundance, we investigated the transcript levels of key mitochondrial proteases, chaperones, and stress markers such as *Clpp*, *Clpx*, *Spg7*, *Afg3l2*, *Hspa9*, *Hspd1*, *Atf3* and *Atf4* in heart tissue from 12-week-old WT, *Lonp1^GKO-HET^*, Cre-Control and *Lonp1^CKO-HET^* mice hearts.

**Figure 4:**
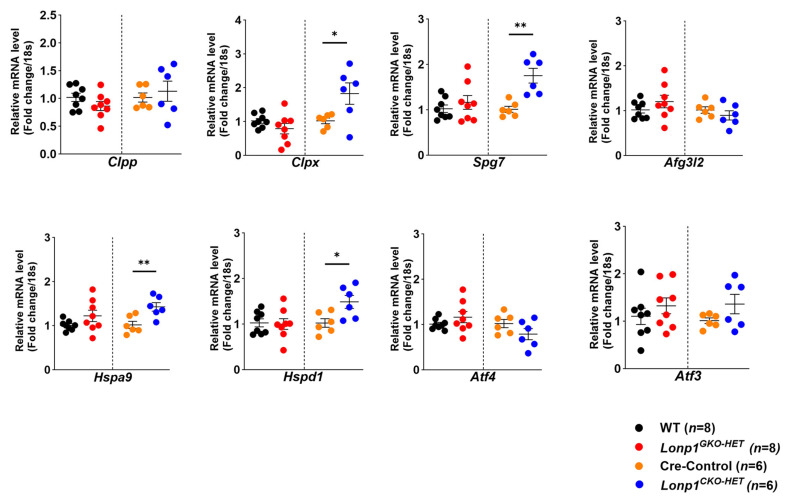
Evaluation of mitochondrial stress response in cardiomyocyte-specific and global *Lonp1* haploinsufficient hearts. Relative mRNA-level fold change in components of mitochondrial ATP dependent proteases like *Clpp*, *Clpx*, *Spg7*, and *Afg3l2* and chaperones like *Hspa9*, *Hspd1*, and integrated stress response markers, *Atf4* and *Atf3* were analyzed in systemic (*Lonp1^GKO-HET^*) (*n* = 8, 4 male and 4 female) and cardiomyocyte-specific (*Lonp1^CKO-HET^*) (*n* = 6, 3 male and 3 female) haploinsufficient along with their respective control mice hearts. Values are represented as mean ± S.E.M. * *p* < 0.05, ** *p* < 0.01 is considered significant by Student’s *t*-test.

Among the investigated transcripts, *Clpp* expression remained unchanged across all groups, while *Clpx* expression was significantly upregulated in *Lonp1^CKO-HET^* hearts compared to their Cre-Control littermates (Figure 4). Similarly, *Spg7* mRNA levels were significantly elevated in *Lonp1^CKO-HET^* hearts, whereas *Afg3l2* levels showed no significant difference among the genotypes (Figure 4). The mitochondrial chaperones *Hspa9* and *Hspd1* were both significantly increased in *Lonp1^CKO-HET^* mice compared to Cre-Controls, with no change in the expression of *Atf4* and *Atf3*, two transcription factors involved in integrated stress response signaling (Figure 4). In contrast, *Lonp1^GKO-HET^* hearts exhibit no measurable differences in the expression of *Hspa9*, *Hspd1*, *Atf4*, and *Atf3* compared to their WT littermates (Figure 4). Together, these findings suggest that the partial loss of *Lonp1*, specifically in cardiomyocytes but not in global tissues, activates a tissue-specific stress response characterized by the selective upregulation of stress-responsive proteases and chaperones, without affecting global integrated stress response markers.

### 3.5. Cardiac-Specific Partial Loss of Lonp1 Alters the Expression of Mitochondrial Biogenesis but Not in Global Lonp1 Haploinsufficiency Hearts

**Figure 5:**
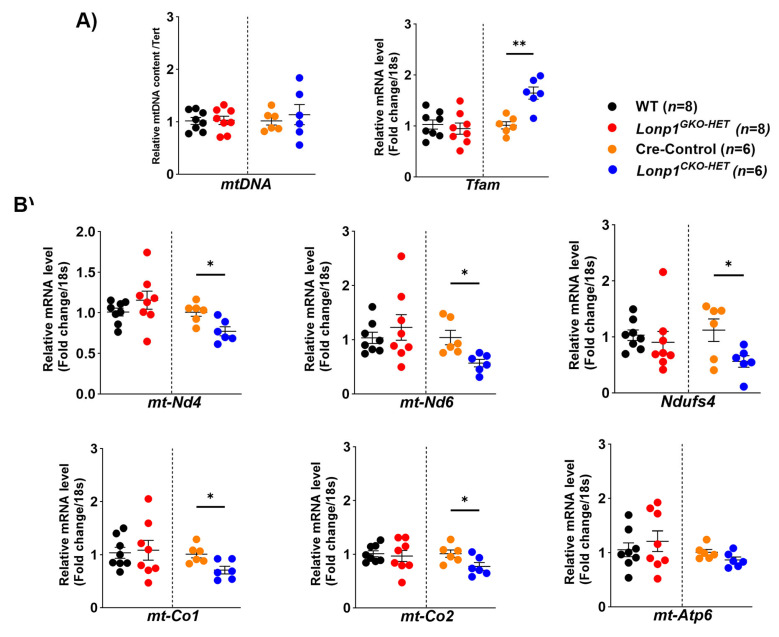
Mitochondrial biogenesis in cardiac-specific and global *Lonp1* haploinsufficient hearts. (**A**) Relative mtDNA content normalized to nDNA (Tert) and relative fold change in the mRNA level expressions of mitochondrial transcription factor A (*Tfam*) in the systemic and cardiomyocyte-specific *Lonp1* haploinsufficient mice hearts and their control littermates. (**B**) Relative fold change in the mRNA level expressions of mitochondrial encoded respiratory complex genes like *mt-Nd4*, *mt-Nd6*, *mt-Co1*, *mt-Co2*, *mt-Atp6* and nuclear encoded *Ndufs4* were analyzed in systemic (*Lonp1^GKO-HET^*) (*n* = 8, 4 male and 4 female) and cardiomyocyte-specific (*Lonp1*^CKO-HET^) (*n* = 6, 3 male and 3 female) heterozygous mice hearts compared with their respective controls. Values are represented as mean ± S.E.M. * *p* < 0.05, ** *p* < 0.01 is considered significant by Student’s *t*-test.

The heart is highly dependent on mitochondrial metabolism, and sustaining a healthy mitochondrial population is of utmost importance for cardiac homeostasis [24]. To investigate whether *Lonp1* haploinsufficiency affects mitochondrial DNA (mtDNA) content and the transcription of mitochondrial genes, we analyzed the relative mtDNA content, expression of mitochondrial transcription factor A (*Tfam*), and selected nuclear- and mtDNA-encoded genes involved in oxidative phosphorylation (OXPHOS). Relative mtDNA content remained unchanged in both *Lonp1^GKO-HET^* and *Lonp1^CKO-HET^* hearts, compared to their respective littermate controls (Figure 5A). In contrast, expression of *Tfam* was significantly upregulated in *Lonp1^CKO-HET^* hearts compared to Cre-Controls (Figure 5A). The expression of *mt-Nd4* and *mt-Nd6*, encoding subunits of Complex I, was significantly reduced in *Lonp1^CKO-HET^* hearts (Figure 5B). Similarly, the expression of nuclear-encoded Complex I subunit *Ndufs4* was also significantly lower in *Lonp1^CKO-HET^* compared to Cre-Control (Figure 5B). We also examined genes encoding subunits of Complex IV and Complex V; *mt-Co1* and *mt-Co2*, which encode cytochrome c oxidase subunits I and II, were significantly downregulated in *Lonp1^CKO-HET^* hearts (Figure 5B). Expression of *mt-Atp6*, a subunit of ATP synthase, showed no significant changes across genotypes (Figure 5B). Together, these findings suggest that cardiac-specific *Lonp1* haploinsufficiency disrupts the transcriptional regulation of key mitochondrial genes involved in biogenesis and oxidative phosphorylation, without altering overall mtDNA content, which is not observed in *Lonp1^GKO-HET^* hearts.

### 3.6. Assessment of Mitochondrial Respiratory Chain Complex Activities in Lonp1 Haploinsufficient Hearts

**Figure 6:**
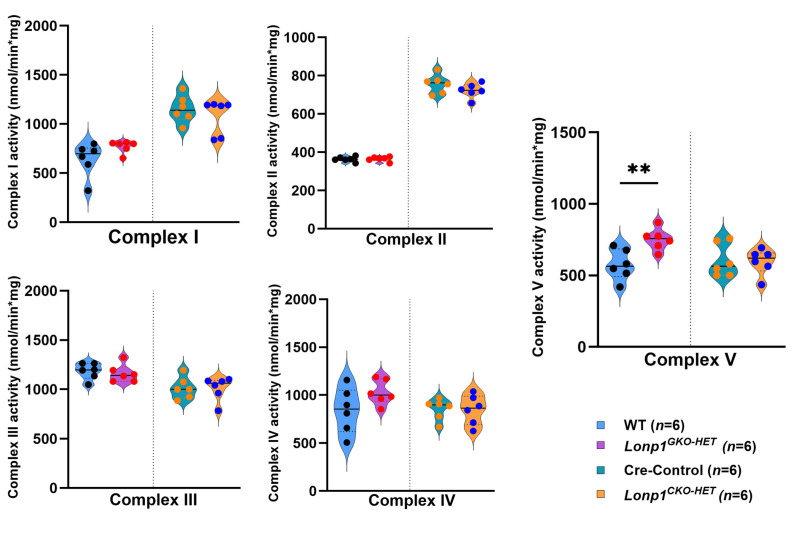
Mitochondrial respiratory chain complex activities in cardiac-specific and global *Lonp1* haploinsufficiency mice hearts. Complex activities were measured spectrophotometrically and expressed as nmol/min per mg of mitochondrial lysate. All the complex activities were averaged from 2 assays from the pooled samples. Complex I activity, measured by NADH oxidation, Complex II activity, assessed by dichlorophenolindophenol reduction, Complex III activity measured by cytochrome c reduction, Complex IV activity, measured by cytochrome c oxidation, and Complex V activity measured by NADH oxidation. Values are represented as mean ± S.E.M. ** *p* < 0.01 is considered significant by Student’s *t*-test.

To determine whether *Lonp1* haploinsufficiency affects the activities of electron transport chain (ETC) Complexes I–V in heart lysates from 12-week-old mice of different genotypes (Figure 6). The activities of Complexes I, II, III, and IV were not significantly different among the groups compared with their respective controls. However, Complex V (ATP synthase) activity was significantly increased in *Lonp1^GKO-HET^* hearts compared to their WT littermates, while Complex V activity in *Lonp1^CKO-HET^* remained comparable to Cre-Control, suggesting a differential compensatory adaptation in systemic versus cardiac-restricted *Lonp1* haploinsufficiency.

### 3.7. Cardiomyocyte-Specific Lonp1 Haploinsufficiency Disrupts Mitochondrial Dynamics

**Figure 7:**
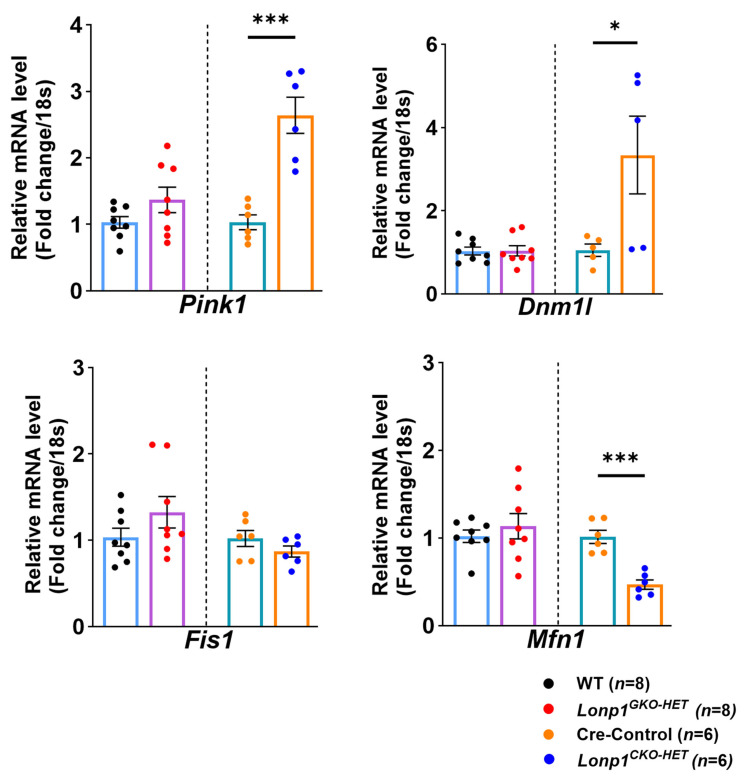
*Lonp1* haploinsufficiency alters mitochondrial dynamics in a cardiac-specific manner. The relative mRNA expression levels of mitophagy-associated transcripts, such as *Pink1*, *Dnm1l*, *Mfn1*, and *Fis1* in *Lonp1^CKO-HET^*, *Lonp1^GKO-HET^*, and their respective control mice. Values are represented as mean ± S.E.M. * *p* < 0.05, *** *p* < 0.001 is considered significant by Student’s *t*-test.

A key regulator of mitochondrial dynamics and quality control genes, like *Pink1*, *Dnm1l*, *Fis1*, and *Mfn1* transcripts, was analyzed in both mouse models to evaluate whether *Lonp1* deficiency impacts mitochondrial dynamics and quality control pathways. Expression of the mitophagy regulator *Pink1* was significantly elevated in *Lonp1^CKO-HET^* hearts compared to controls (Figure 7). Among fission-related genes, *Dnm1l* was induced considerably in *Lonp1^CKO-HET^* hearts, while *Fis1* expression remained unchanged across all genotypes (Figure 7). The mitochondrial fusion gene *Mfn1* was significantly downregulated in *Lonp1^CKO-HET^* compared to their Cre-Control littermates, suggesting a shift in the balance between fission and fusion. In contrast, no significant changes in the expression of these mitochondrial dynamic markers were noted in the global *Lonp1* haploinsufficiency hearts.

## 4. Discussion

Our findings demonstrate that cardiomyocyte-specific haploinsufficiency of *Lonp1* leads to early signs of cardiac and mitochondrial stress, whereas global haploinsufficiency does not elicit comparable changes. A multifunctional mitochondrial LONP1 is crucial for cellular proteostasis, whereas homozygous deletion of *Lonp1* in mice results in embryonic lethality [8,21]. Therefore, we have employed global heterozygous mice, which are born normal and live similarly to wild-type mice, and compared them with cardiac-specific heterozygous mice [16]. Our results highlight the unique vulnerability of post-mitotic cardiomyocytes to impaired mitochondrial proteostasis and suggest that systemic, extra-cardiac mitochondrial stress resulting from *Lonp1* haploinsufficiency may buffer against such localized stress, possibly through compensatory inter-organ signaling.

The differential mitochondrial stress response between the two models further underscores this tissue-specific sensitivity. *Lonp1^CKO-HET^* hearts exhibited robust transcriptional upregulation of mitochondrial stress markers, including *Clpx*, *Spg7*, *Hspa9*, and *Hspd1*, without alterations in integrated stress response factors like *Atf4* and *Atf3*. A similar stress response, serving as an adaptive mechanism, has been observed in other models of *Lonp1* inhibition [25]. The activation of the mitochondrial unfolded protein response (UPR^mt^) mitigates mitochondrial dysfunction and preserves cellular viability in the absence of a broader integrated stress response, suggesting a conserved protective role for LONP1-regulated proteostasis across tissues [25]. Conversely, *Lonp1^GKO-HET^* hearts did not show similar transcriptional alterations, suggesting that systemic *Lonp1* reduction may activate compensatory mechanisms in extra-cardiac tissues that mitigate mitochondrial stress specifically in the heart. This lack of stress response in the global model implies a potential inter-organ communication axis that buffers cardiac mitochondria against proteostasis imbalance, possibly through circulating factors or metabolic adaptations [26]. These findings underscore the concept that localized mitochondrial stress, such as in post-mitotic cardiomyocytes, cannot always be recapitulated by global gene dosage reduction, highlighting the heart’s unique susceptibility and limited capacity to compensate for intrinsic mitochondrial dysfunction.

Mitochondrial biogenesis and OXPHOS gene expression were also differentially affected between the two models of *Lonp1* haploinsufficiency. Interestingly, although mtDNA content remained unchanged, the significant upregulation of *Tfam* in *Lonp1^CKO-HET^* hearts may reflect a pre-emptive compensatory mechanism aimed at enhancing mitochondrial biogenesis and transcriptional capacity in response to early mitochondrial stress, consistent with an adaptive attempt to preserve mitochondrial function. This was accompanied by a notable downregulation of mitochondrial-encoded subunits of Complex I (*mt-Nd4*, *mt-Nd6*) and Complex IV (*mt-Co1*, *mt-Co2*), suggesting impaired mitochondrial respiratory capacity in cardiomyocytes under proteostatic stress. These findings are consistent with previous reports indicating that LONP1 is required for maintaining mtDNA integrity and mitochondrial transcription, and its loss can lead to defective respiratory chain complex assembly [21]. It has been demonstrated that LONP1 reduction in different cell types, such as fibroblasts and neonatal rat ventricular myocytes, led to decreased levels of respiratory chain function and mitochondrial membrane potential, implicating LONP1 as a key regulator of mitochondrial protein homeostasis and energetic function, consistent with the observation in *Lonp1^CKO-HET^* hearts [27].

In contrast, *Lonp1^GKO-HET^* hearts did not exhibit similar transcriptional changes in OXPHOS genes. Surprisingly, they showed an increase in Complex V activity, suggesting a systemic adaptation that enhances mitochondrial ATP production. This finding aligns with evidence that moderate mitochondrial stress can trigger adaptive responses via mitochondrial hormesis (mitohormesis), where mild impairment leads to compensatory upregulation of mitochondrial function in distant tissues [19,20]. Although we did not directly assess the origin of systemic compensation in this study, our findings support the concept that *Lonp1* haploinsufficiency in extra-cardiac tissues can trigger adaptive responses that mitigate cardiac mitochondrial dysfunction. Previous studies have shown that mitochondrial perturbations in metabolically active organs such as skeletal muscle, liver, and adipose tissue can initiate the release of mitokines, a hormone-like signals such as FGF21 and GDF15, which exert systemic effects on mitochondrial biogenesis, oxidative stress, and cellular metabolism in distant organs, including the heart [13,14,15,26,28]. In particular, FGF21, which is secreted by the liver and skeletal muscle in response to mitochondrial stress, has been shown to improve mitochondrial function and reduce inflammation in cardiac tissues [28]. Likewise, GDF15 is another stress-responsive cytokine that mediates metabolic adaptation in response to mitochondrial dysfunction and has been implicated in protective cardiac signaling [26]. Therefore, it is possible that *Lonp1^GKO-HET^* mice, which exhibit moderate mitochondrial proteostatic imbalance in multiple organs, activate such mitokine signaling pathways that confer endocrine-mediated cardio protection.

Moreover, it has been demonstrated that mitochondrial stress in skeletal muscle can upregulate antioxidant defense and reduce cardiac oxidative stress, potentially through altered nutrient flux or humoral signaling [26]. The lack of mitochondrial stress response activation in *Lonp1^GKO-HET^* hearts, combined with increased Complex V activity, supports the concept of a systemic metabolic preconditioning effect, a phenomenon consistent with mitohormesis, where mild stress induces adaptive resilience [19,29,30]. While the specific organ responsible for initiating the compensatory response remains to be determined, skeletal muscle and liver are strong candidates due to their high mitochondrial density, secretory profile, and established roles in mitokine production. Notably, the lack of such a compensatory response in *Lonp1^CKO-HET^* hearts underscores the tissue-autonomous vulnerability of cardiomyocytes to mitochondrial proteostasis imbalance and further supports the notion that systemic mitochondrial stress may paradoxically buffer local dysfunction through endocrine or metabolic crosstalk.

Furthermore, alterations in mitochondrial dynamics were evident only in *Lonp1^CKO-HET^* hearts, which displayed significant upregulation of *Pink1* and *Dnm1l*, markers of mitophagy and mitochondrial fission, respectively, alongside downregulation of the fusion regulator *Mfn1*. These changes reflect an adaptive mitochondrial quality control (QC) mechanism that promotes organelle turnover and remodeling in response to proteostatic stress. This pattern is consistent with prior reports that *Lonp1* deficiency leads to the activation of mitophagy via the Pink1/Parkin signaling pathway [28,31]. The downregulation of *Mfn1*, which is the major driver of mitochondrial fusion, is also in line with the observed suppression of fusion machinery, which facilitates mitochondrial fragmentation in its absence; however, whether that helps in the removal of damaged mitochondria via mitophagy is unclear [32,33]. In contrast, these mitochondrial dynamic regulators remained unchanged in *Lonp1^GKO-HET^* hearts, suggesting that systemic reduction of *Lonp1* does not elicit the same local mitochondrial stress activation in the heart. This reinforces the concept that systemic mitochondrial proteostasis disruption is buffered by extra-cardiac tissues, preventing maladaptive mitochondrial remodeling within the myocardium. Such buffering may be mediated through systemic metabolic adaptations or mitokine signaling, as proposed in models of mild mitochondrial dysfunction, where whole-body adaptations preserve organ-specific mitochondrial integrity [28,34].

In summary, our study reveals that the heart’s reliance on efficient mitochondrial proteostasis makes it uniquely sensitive to *Lonp1* loss when not supported by systemic compensatory mechanisms. While systemic *Lonp1* haploinsufficiency appears to maintain cardiac function through unidentified protective inter-organ mechanisms, as targeted cardiac loss results in early functional decline and mitochondrial stress. These findings underscore the importance of tissue context in mitochondrial matrix protein quality control, suggesting that enhancing systemic mitochondrial proteostasis could be a novel therapeutic strategy for protecting the heart in mitochondrial disorders. Our study offers important translational insights into mitochondrial-targeted therapies for cardiac disease. We show that systemic *Lonp1* haploinsufficiency triggers mild mitochondrial stress and adaptive responses (mitohormesis), potentially preconditioning the heart against dysfunction. In contrast, cardiomyocyte-specific *Lonp1* reduction leads to a trend towards cardiac dysfunction without systemic compensation, highlighting the heart’s reliance on intrinsic LONP1 activity. These findings suggest that systemic modulation of LONP1 may be a safer and more flexible therapeutic approach than direct cardiac targeting, offering a novel strategy to enhance cardiac resilience through inter-organ mitochondrial signaling.

Our study focused on 12-week-old mice to assess early mitochondrial stress responses and cardiac function before potential age-associated compensatory changes. While this time point provides insights into early pathophysiological events, it remains unknown whether *Lonp1* haploinsufficiency leads to progressive deterioration over time. Previous studies suggest that mitochondrial proteostasis deficits can accumulate with age [8,22,25], raising the possibility that older *Lonp1^CKO-HET^* mice may exhibit more severe cardiac remodeling or dysfunction. However, longitudinal or age-cohort studies are underway in these specific models.

### 4.1. Limitations

Our study has several limitations. Firstly, while our findings suggest protective inter-organ communication in systemic *Lonp1* haploinsufficiency, we did not directly identify the source organs or molecular mediators (e.g., mitokines or metabolic hormones) responsible for this compensation. However, based on existing literature, we predict that skeletal muscle and liver could be the primary contributors through the secretion of FGF21 and GDF15 in response to mitochondrial stress. Secondly, the analyses were limited to young adult mice under baseline conditions, and it remains unclear how these models respond to aging or cardiac stress. Lastly, potential sex-specific effects were noted but not fully explored due to limited sample size, warranting further investigation in future studies.

### 4.2. Future Direction

Future studies should focus on identifying the specific extra-cardiac tissues and signaling pathways involved in this systemic compensation. Additionally, exploring whether enhancing mitochondrial proteostasis in peripheral organs can confer cardioprotection under pathological conditions may open new avenues for treating mitochondrial cardiomyopathies. Furthermore, evaluating the plasma levels of mitokines such as FGF21 and GDF15 and determining their expression profiles in skeletal muscle and liver in *Lonp1^GKO-HET^* mice could offer insights into the inter-organ crosstalk mechanism. Incorporating ultrastructural analyses using transmission electron microscopy (TEM) in the future will be valuable for directly visualizing mitochondrial morphology (e.g., swelling, fragmentation, cristae structure) and validating the transcriptional and functional findings observed in *Lonp1* haploinsufficient hearts. Future studies incorporating omics-based approaches, including proteomics, metabolomics, and transcriptomics, will be essential to identify circulating factors and molecular networks that mediate inter-organ crosstalk in response to *Lonp1* haploinsufficiency.

## 5. Conclusions

In conclusion, our study demonstrates that cardiomyocyte-specific haploinsufficiency of *Lonp1* leads to mitochondrial stress and a trend towards cardiac dysfunction. In contrast, systemic *Lonp1* haploinsufficiency elicits no such pathology, suggesting a protective role of inter-organ compensatory mechanisms. The heart’s unique vulnerability to mitochondrial proteostasis disruption, especially in post-mitotic cardiomyocytes, underscores the critical importance of tissue context in maintaining mitochondrial function. The absence of mitochondrial stress responses and maladaptive remodeling in globally haploinsufficient mice highlights the potential for systemic buffering, possibly via mitokine signaling or metabolic adaptations. These findings not only emphasize the indispensable role of LONP1 in sustaining cardiac mitochondrial homeostasis but also reveal a novel paradigm in which enhancing systemic mitochondrial quality control may offer therapeutic benefits in cardiac disease settings characterized by mitochondrial dysfunction.

## Data Availability

Raw data for the original contributions presented in this study are included in the Supplementary Materials. Further inquiries can be directed to the corresponding author.

## Supplementary Materials

The following supporting information can be downloaded at: https://www.mdpi.com/article/10.3390/biom15081159/s1, Figure S1: Gross morphology assessment of *Lonp1^GKO-HET^* and *Lonp1^CKO-HET^* female mice; Figure S2: Histological evaluation of cardiac remodeling in *Lonp1^GKO-HET^* and *Lonp1^CKO-HET^* mice; Table S1: List of TaqMan gene expression assays; Table S2: Raw values for qPCR, echocardiography, histology, and complex activity assays.

## Abbreviations

ATP	Adenosine Triphosphate
Atf3	Activating Transcription Factor 3
Atf4	Activating Transcription Factor 4
CKO	Conditional Knockout
Clpp	Caseinolytic Mitochondrial Matrix Peptidase Proteolytic Subunit
Clpx	Caseinolytic Mitochondrial Matrix Peptidase Chaperone Subunit
CO	Cardiac Output
Cre	Cre Recombinase
DCPIP	2,6-Dichlorophenolindophenol
Dnm1l	Dynamin-1-like Protein (Drp1)
EF	Ejection Fraction
ETC	Electron Transport Chain
Fis1	Mitochondrial Fission 1 Protein
FS	Fractional Shortening
GDF15	Growth Differentiation Factor 15
GKO	Global Knockout
HE	Hematoxylin and Eosin
Hspa9	Heat Shock Protein Family A Member 9 (Mortalin)
Hspd1	Heat Shock Protein Family D Member 1 (HSP60)
HW/TL	Heart Weight to Tibia Length
LV	Left Ventricle
LV mass	Left Ventricular Mass
LVsD	Left Ventricular Systolic Diameter
LVdD	Left Ventricular Diastolic Diameter
LVAW	Left Ventricular Anterior Wall
LVPW	Left Ventricular Posterior Wall
LONP1	Lon Protease 1
mPQC	Mitochondrial Protein Quality Control
Mfn1	Mitofusin 1
Mitohormesis	Mitochondrial Hormesis
mt-Co1	Mitochondrial Cytochrome c Oxidase Subunit 1
mt-Co2	Mitochondrial Cytochrome c Oxidase Subunit 2
mt-Nd4	Mitochondrial NADH Dehydrogenase 4
mt-Nd6	Mitochondrial NADH Dehydrogenase 6
mtDNA	Mitochondrial DNA
NADH	Nicotinamide Adenine Dinucleotide (reduced form)
Ndufs4	NADH Dehydrogenase [Ubiquinone] Iron-Sulfur Protein 4
OXPHOS	Oxidative Phosphorylation
PCR	Polymerase Chain Reaction
Pink1	PTEN-Induced Kinase 1
qRT-PCR	Quantitative Real-Time PCR
RT-PCR	Reverse Transcription PCR
Spg7	Paraplegin
SV	Stroke Volume
Tfam	Transcription Factor A, Mitochondrial
Tert	Telomerase Reverse Transcriptase
UPRmt	Mitochondrial Unfolded Protein Response
