# Supplementary material for: Systemic Lonp1 Haploinsufficiency Mitigates Cardiac Mitochondrial Dysfunction Induced by Cardiomyocyte-Specific Lonp1 Haploinsufficiency via Potential Inter-Organ Crosstalk
Source: Biomolecules. 2025 Aug 13;15(8):1159. doi: 10.3390/biom15081159 (PMC12384522; doi:10.3390/biom15081159)
Supplement: Supplementary file 1 [file biomolecules-15-01159-s001.zip › Fig S1 and S2.pdf]

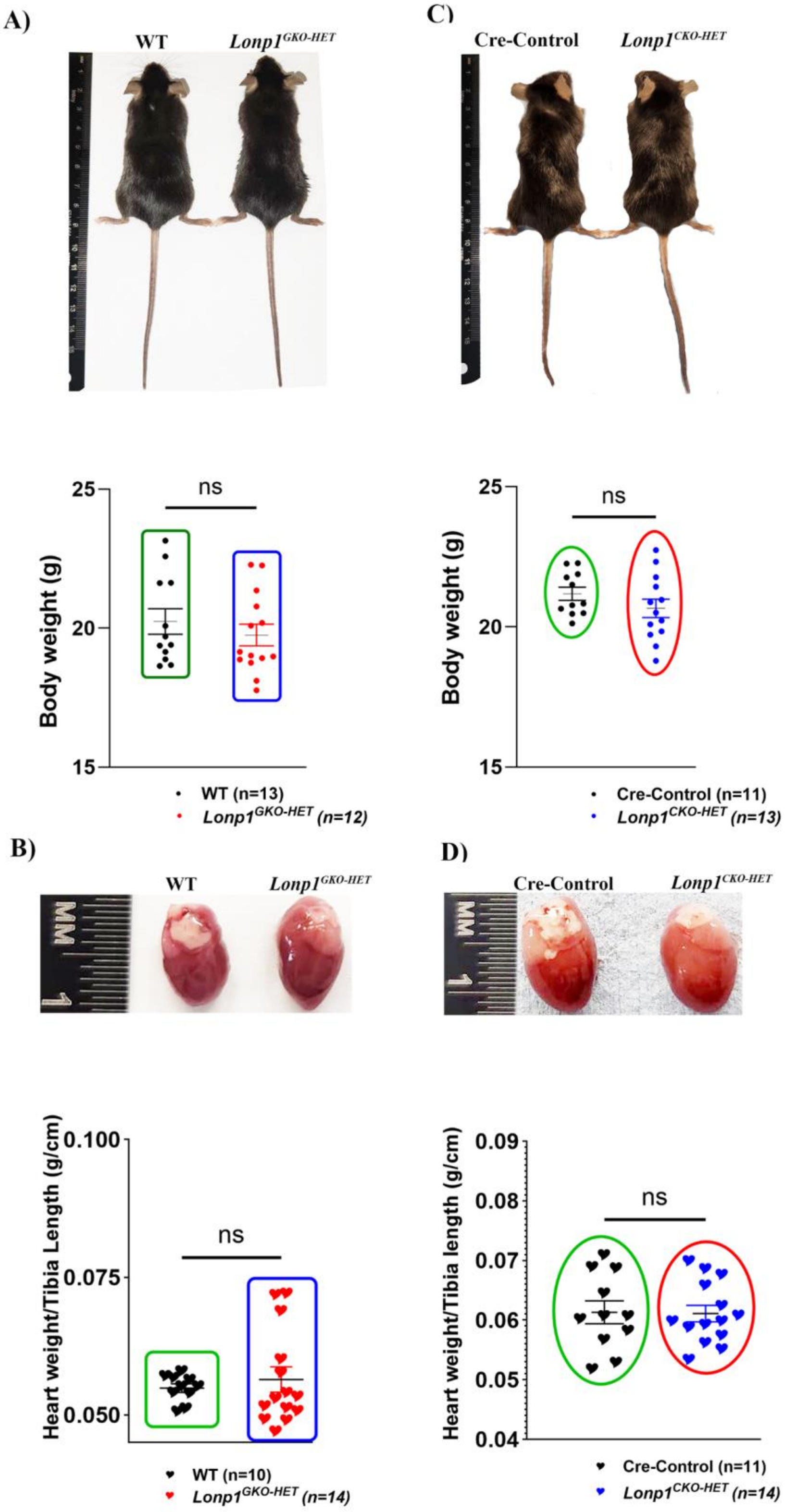

Figure S1. Gross morphology assessment of *Lonp1*$^{GKO\text{-}HET}$ and *Lonp1*$^{CKO\text{-}HET}$ female mice: **(A)** Gross views of global heterozygous (*Lonp1*$^{GKO\text{-}HET}$) and wild-type (WT) littermates of female mice, with the bottom panel showing scattered dot plots of corresponding body weight. **(B)** Corresponding images of excised hearts with the bottom scatter plot showing their heart weight to tibial length (HW/TL). **(C)** Gross dorsal views of cardiac-specific heterozygous (*Lonp1*$^{CKO\text{-}HET}$) and Cre-Control littermates of female mice, with the bottom panel showing scattered dot plots of corresponding body weight. **(D)** Corresponding images of excised hearts with a bottom scatter plot showing their heart weight to tibial length (HW/TL). Data represent mean ± SEM (n = 10–14 per group). Students' unpaired t-test detected no significant difference either in the body weight or the heart weight in both models. "ns" indicates non-significant differences.

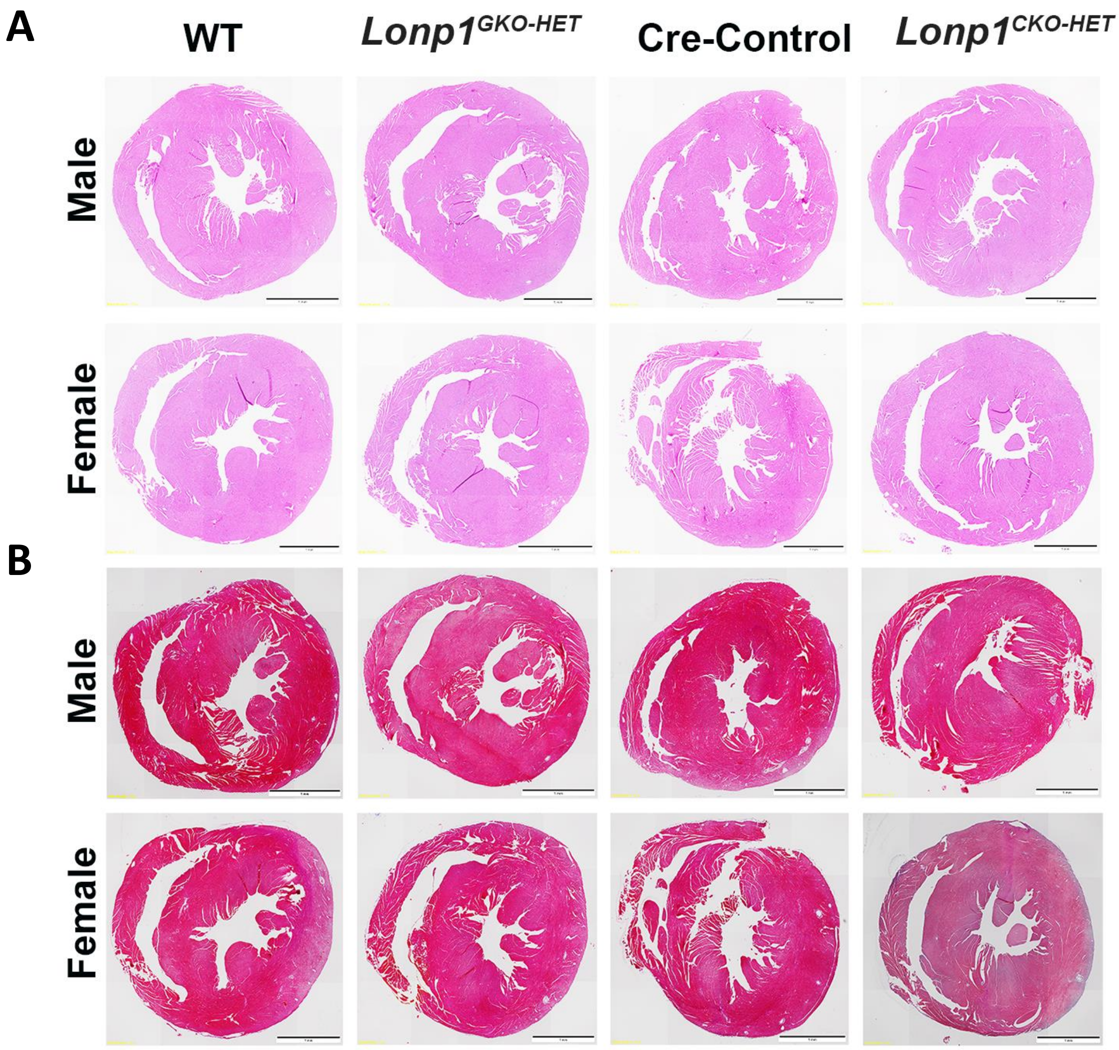

Figure S2. Histological evaluation of cardiac remodeling in *Lonp1*$^{GKO\text{-}HET}$ and *Lonp1*$^{CKO\text{-}HET}$ mice. (**A**) Representative transverse sections from male and female global heterozygous (*Lonp1*$^{GKO\text{-}HET}$), wild-type (WT), and cardiomyocyte-restricted heterozygous (*Lonp1*$^{CKO\text{-}HET}$) and Cre-Control hearts stained with hematoxylin–eosin (H and E, upper panel). **(B)** Representative transverse sections from male and female global heterozygous (*Lonp1*$^{GKO\text{-}HET}$), wild-type (WT), and cardiomyocyte-restricted heterozygous (*Lonp1*$^{CKO\text{-}HET}$) and Cre-Control hearts stained Masson's trichrome. Images are shown at 10 × (scale bar = 1mm).
