# Supplementary material for: Systemic Lonp1 Haploinsufficiency Mitigates Cardiac Mitochondrial Dysfunction Induced by Cardiomyocyte-Specific Lonp1 Haploinsufficiency via Potential Inter-Organ Crosstalk
Source: Biomolecules. 2025 Aug 13;15(8):1159. doi: 10.3390/biom15081159 (PMC12384522; doi:10.3390/biom15081159)
Supplement: Supplementary file 1 [file biomolecules-15-01159-s001.zip › Table S1.pdf]

Table S1. List of TaqMan gene expression assays

**Gene**	**Assay ID**
BioRAD	
Lonp1	qMmuCIP0030530
Clpp	qMmuCIP0028131
Clpx	qMmuCEP0052640
Spg7	qMmuCEP0062183
Afg3l2	qMmuCIP0033299
Hspa9	qMmuCIP0034670
HSpd1	qMmuCEP0042787
Atf4	qMmuCEP0056683
Atf3	qMmuCEP0035769
Tfam	qMmuCEP0053627
Mt-Nd4	qMmuCEP0060082
Mt-Nd6	qMmuCEP0062889
Ndufs4	qMmuCEP0054292
Mt-Co1	qMmuCEP0043400
Mt-Co2	qMmuCEP0037258
Mt-Atp6	qMmuCEP0060077
Pink1	qMmuCEP0035988
Dnm1l	qMmuCEP0034029
Fis1	qMmuCEP0034407
Mfn1	qMmuCEP0034198
**Applied Biosystems**	
18S	2308044
Tert	4458373
